# Decisive Effects of Life Stage on the Gut Microbiota Discrepancy Between Two Wild Populations of Hibernating Asiatic Toads (*Bufo gargarizans*)

**DOI:** 10.3389/fmicb.2021.665849

**Published:** 2021-08-03

**Authors:** Xiaowei Song, Jingwei Zhang, Jinghan Song, Yuanyuan Zhai

**Affiliations:** ^1^College of Life Sciences, Xinyang Normal University, Xinyang, China; ^2^Institute for Conservation and Utilization of Agro-Bioresources in Dabie Mountains, Xinyang Normal University, Xinyang, China; ^3^Chengdu Institute of Biology, Chinese Academy of Sciences, Chengdu, China; ^4^Hospital of Xinyang Normal University, Xinyang Normal University, Xinyang, China

**Keywords:** amphibian, driving factor, gut microbiota, hibernation, Proteobacteria, small intestine, 16S rRNA

## Abstract

Until now, the effects of driving factors on the gut microbiota of amphibians are still mostly confounded. Due to a long-term fasting, hibernating amphibians are ideal experimental materials to explore this question. In this study, we characterized the small intestine microbiota of adult hibernating Asiatic toads (*Bufo gargarizans*) collected from two geographical populations using 16S rRNA amplicon sequencing technique and evaluated the effects of non-dietary factors (e.g., sex and host genetic background). Proteobacteria (0.9196 ± 0.0892) was characterized as the most dominant phylum in the small gut microbiota of hibernating Asiatic toads, among which five core OTUs were identified and three were classified into *Pseudomonas*. In view of the coincidence between the dominant KEGG pathways (such as the two-component system) and *Pseudomonas*, *Pseudomonas* appeared to be a key adaptor for small gut microbiota during hibernation. Furthermore, we detected a greater discrepancy of gut microbiota between geographical populations than between sexes. Both sex and host genetic background showed a minor effect on the gut microbiota variation. Finally, life stage was determined to be the decisive factor driving the gut microbiota discrepancy between populations. However, a large proportion of the gut microbiota variation (∼70%) could not be explained by the measured deterministic factors (i.e., sex, location, body length, and routine blood indices). Therefore, other factors and/or stochastic processes may play key roles in shaping gut bacterial community of hibernating amphibians.

## Introduction

Gut microbial community acts as a key regulator in host normal physiological activities and health maintenances (Sommer and Bäckhed, 2013; Feng et al., 2018; Simon et al., 2019). The structure and function of intestinal microbial community depend on both extrinsic factors (e.g., diet and habitat) and intrinsic factors (e.g., life stage and genetic background) (Sommer and Bäckhed, 2013; Jiménez and Sommer, 2017; Feng et al., 2018). The mutualistic symbionts coevolve with hosts at a long-term timescale, thereby they may exhibit a host phylogenetic signal, i.e., phylosymbiosis (McFall-Ngai et al., 2013; Brooks et al., 2016; Amato et al., 2019). As cold-blooded vertebrates, amphibians (such as frogs, toads, or salamanders) have gilled aquatic larvae and air-breathing adults, and they have intermediate characters between fishes and reptiles. The key intrinsic and extrinsic factors of amphibians assemble unique gut microbiota, i.e., gut microbial community. For instance, the gut microbiota of adult amphibians is more similar to that of mammals rather than fishes (Kohl et al., 2013; Colombo et al., 2015). By contrast, the gut bacterial community of amphibian tadpoles is more similar to that of fishes (Kohl et al., 2013; Vences et al., 2016; Chai et al., 2018).

Previous researches mainly focus on the effects of individual factors on the gut microbiota (i.e., bacterial community) of amphibians using culture-independent methods (Kohl et al., 2013; Bletz et al., 2016; Chang et al., 2016; Kohl and Yahn, 2016; Weng et al., 2016; Zhang et al., 2016). In recent years, more efforts were devoted to the compound effects of multiple factors on the gut microbiota of amphibians (Vences et al., 2016; Weng et al., 2016, 2017; Huang et al., 2017; Warne et al., 2017; Knutie et al., 2018; Tong et al., 2019b). For instance, the gut microbiota of amphibian larvae possesses a certain degree of species specificity, but the interspecific variation is probably subdominant (Vences et al., 2016; Warne et al., 2017). At the population scale, nevertheless, the relationship between host genetic background and the gut microbiota of amphibians is still not clear.

Amphibians generally enter a hibernation stage in winter to counteract the energy limitation in adverse circumstances, such as cold temperature and food shortage. The Asiatic toad (*Bufo gargarizans*), a widely distributed true toad in China, prefers overwintering in subaqueous or subterraneous habitats of ponds, lakes, or rivers (Fei et al., 2012). Since higher-latitude regions generally maintain a longer cold temperature in winter than lower-latitude regions, the hibernation timespan of Asiatic toads shows a remarkable latitude-correlated variation (3–8 months), i.e., longer hibernation stage in higher-latitude regions. The structure of amphibian gut microbiota is significantly remodeled along with dramatic changes in physiological and metabolic activities during hibernation (Banas et al., 1988; Costanzo and Lee, 2013; Weng et al., 2016; Wiebler et al., 2018). Due to a long-term fasting, hibernating amphibians are ideal experimental materials to explore the effects of non-dietary factors (e.g., genetic background) on the gut microbiota (Tong et al., 2019a). In this study, we explored the gut microbiota of adult hibernating Asiatic toads collected from two geographical locations. The aim was to evaluate the relative effects of multiple intrinsic factors (i.e., sex, life stage, health condition, and genetic background) on the gut microbiota of amphibians.

## Materials and Methods

### Preparation of Experimental Specimens

We acquired two wild populations of hibernating Asiatic toads (*B. gargarizans*) from fishermen, who collected these toads in Ji Canal of Tianjin City (TJ population, *n* = 22) and Luoma Lake of Xuzhou City (XZ population, *n* = 23) during the winter in 2014. The two populations located in a similar longitude possessed a geographical distance of about 600 km (Supplementary Figure 1). The ecological systems of Ji Canal and Luoma Lake tend to be similarly affected by human activities, e.g., agriculture (Chen et al., 2000; Liu et al., 2021). These toads were placed into drinking water (5–10°C) before sacrificed by pithing the brain and spinal cord. After double pithing, we immediately sampled the experimental specimens (e.g., cardiac blood and small intestine) of each toad. Specifically, the cardiac blood was collected into anticoagulant tubes by using aseptic injectors. The small intestine cut off by sterile scissors was stored in a −80°C refrigerator until DNA extraction. For this research, we applied 25 sample individuals, among which 14 belonged to the TJ population and 11 belonged to the XZ population (Supplementary Table 1). All procedures used in this study were approved by the Animal Care and Use Committee of Xinyang Normal University.

### Measurement of Intrinsic Factors for Hibernating Asiatic Toads

The intrinsic factors measured for hibernating toads consisted of sex, life stage, health condition, and genetic background. Specifically, we identified the sex of toads by checking the sexual characteristics (i.e., nuptial pad and reproductive system). Although the body size of Asiatic toads presents a latitude- and sex-dependent variation, body size is an effective indicator for age (i.e., life stage) (Yu and Lu, 2013). We evaluated the life stage of toads by measuring five morphological traits, i.e., body length (i.e., snout–vent length), body mass, body mass/body length (BM:BL), eye space, and nasal space. Subsequently, the health condition was evaluated through routine blood test (RBT) for lactate dehydrogenase, creatine kinase, alanine aminotransferase, aspartate aminotransferase, total protein, albumin, globulin, and alkaline phosphatase. These RBT factors were measured using the autochemistry analyzer CS-600B and corresponding kits (Dirui Industrial Inc., Changchun, China). Finally, we evaluated the host genetic divergence by using mitochondrial DNA (mtDNA: cytb and D-loop) and microsatellite [i.e., simple sequence repeat (SSR)] markers. The mtDNA and SSR markers were amplified by using the primers designed in previous studies (Supplementary Table 2). The polymerase chain reaction (PCR) products of mtDNA were sequenced by using ABI 3730xl DNA analyzer (Applied Biosystems, Bedford, MA, United States) in a commercial company (GenScript Biotech Corporation, Nanjing, China). The sequences have been deposited into CNGB sequence archive (CNSA) of China National GeneBank DataBase (CNGBdb) with project number CNP0001473 (Guo et al., 2020). We genotyped the PCR samples of SSRs by means of polyacrylamide gel electrophoresis. We deduced the genetic relationship between sample individuals using mtDNA and SSRs, respectively. Steps for the mtDNA process were listed as follows: (i) multiple sequence alignment was executed on cytb and D-loop fragments with ClustalW (version 1.4) embedded in BioEdit (version 7.2.5), respectively (Thompson et al., 1994; Hall, 1999); (ii) haplotype sequences of concatenated cytb and D-loop fragments were generated by DnaSP (version 5.10) with gap consideration (Librado and Rozas, 2009); and (iii) maximum likelihood (ML) and Bayesian phylogenetic trees of these haplotypes were reconstructed by using RAxML (version 8.2.10) and MrBayes (version 3.2), respectively (Ronquist et al., 2012; Stamatakis, 2014). In phylogenetic reconstructions, cytb and D-loop fragments were treated as separate partitions. A GTR + G model was applied to each partition in both ML and Bayesian tree reconstruction. The number of bootstrap runs was set to 1,000 in ML tree reconstruction. Two independent runs and four chains in each run were applied for the Bayesian inference. The posterior probability of Bayesian trees was calculated from tree spaces generated by 0.5 burnin fraction after 10,000,000 generations. As for SSRs, we used R package “vegan” (version 2.5-2) to calculate the between-individual Bray–Curtis dissimilarity.

### Metagenomic DNA Extraction, 16S rRNA Gene Amplicon Sequencing, and Operational Taxonomic Unit Clustering

We utilized liquid nitrogen to grind and homogenize the small intestine of each toad, and then applied the phenol–chloroform method (Song et al., 2018) to extract metagenomic DNA from each small intestine (Supplementary Table 1). The DNA quality was determined using agarose gel electrophoresis or NanoVue Plus Spectrophotometer (GE Healthcare Inc., Princeton, NJ, United States). DNA samples were stored in a −20°C refrigerator for 16S rRNA gene amplification. The PCR conditions for 16S rRNA gene (hypervariable regions: V3–V4) and following MiSeq sequencing were performed in a commercial company (Biobit Biotech Inc., Chengdu, China). The detailed procedures were described previously (Xu et al., 2020). The V3–V4 regions of bacterial 16S rRNA genes were amplified using the universal primer pair 341F—CCTACGGGNGGCWGCAG and 805R—GACTACHVGGGTATCTAATCC (Klindworth et al., 2012). The raw reads of 16S rRNA genes can be found in the CNSA of CNGBdb with project number CNP0001644 (Guo et al., 2020). The paired-end reads were merged by FLASH software (version 1.2.11) with a minimum overlap of 20 base pairs (bp) and a maximum overlap of 250 bp (Magoč and Salzberg, 2011). The quality control for primer-filtered reads was achieved using VSEARCH software (version 2.4.4) (Rognes et al., 2016). Specifically, those sequences with more than nine expected errors or zero ambiguous bases were discarded. Sequences with less than 300 bases were also discarded. In addition, the chimeras were detected using uchime_denovo with the parameter “–abskew 1.” Subsequently, we clustered the dereplicated quality-controlled sequences into operational taxonomic units (OTUs) using the VSEARCH software with the identity threshold of 97% (Rognes et al., 2016). The chimeras were detected again with the above same procedure. The OTU table was generated with usearch_global program with parameters “–id 0.97 –maxrejects 0 –maxaccepts 2.”

### Structural and Functional Annotation of Gut Microbiota

We profiled gut microbiota structures using the QIIME2 toolbox (version 2018.6) (Bolyen et al., 2019). Specifically, the core OTUs in a group were detected in each individual of this group. Venn diagrams^1^ were depicted to show the shared OTUs between groups. The MAFFT method in the alignment plugin was used to align OTU representative sequences (Katoh and Standley, 2013), and then the phylogenetic tree of these sequences was reconstructed using the fasttree method in the phylogeny plugin (Price et al., 2010). Taxonomic classification of the OTU representative sequences was achieved using a scikit-learn classifier (confidence threshold = 0.8) in terms of Greengenes (version 13.8) (Pedregosa et al., 2011; McDonald et al., 2012). Stack bars were depicted to show taxonomic compositions of gut microbiota. To annotate the function of gut microbiota, we created a compatible OTU table for PICRUSt in terms of Greengenes (version 13.5) using usearch_global program with parameters “–id 0.97 –maxrejects 0 –maxaccepts 2,” and then predicted KEGG (Kyoto Encyclopedia of Genes and Genomes) orthology (KO) and KEGG pathways by using PICRUSt (online Galaxy version 1.1.1^2^) (Langille et al., 2013).

To statistically analyze α diversity (i.e., observed OTUs, Shannon, Pielou’s evenness, and Faith’s PD) and β diversity indices (i.e., Bray–Curtis, Jaccard, weighted UniFrac, and unweighted UniFrac), we rarefied the OTU table to the lowest sampling depth (i.e., 6,301). Moreover, we tested whether the sampling depth was appropriate by calculating Spearman correlations of α and β diversity indices between 10 iteratively rarefied OTU tables. The α and β diversity indices of the samples showed high Spearman correlation coefficients between iterative rarefied OTU tables at a sampling depth of 6,000 and 6,301, respectively (Supplementary Figure 2). The α diversity and relative abundances of taxa or KEGG pathways in groups were shown as “mean ± SD (standard deviation)” unless otherwise stated.

### Statistical Analysis for the Structure and Function of Gut Microbiota

First, we utilized two-way ANOVA (linear model and type III sum of squares) to test the effects of sex and location on α diversity indices and intrinsic factors. Two-way PERMANOVA was carried out to test the effects of sex and location on Bray–Curtis and Jaccard distance matrices of the rarefied OTU table. The permutation number was set to be 9,999. These statistical analyses were executed using R project (version 3.6.1). We identified taxonomic markers of gut microbiota in two sexes or populations using the LEfSe method (online Galaxy version 1.0^3^) (Segata et al., 2011). Per-sample sum was normalized to one million as the algorithm designers recommend. Both α values for the factorial Kruskal–Wallis test among classes and the pairwise Wilcoxon test between subclasses were set to 0.01. The threshold on the logarithmic LDA score for discriminative features was set to 2.0. All-against-all strategy was executed for multiclass analysis. We used the STAMP software (version 2.1.3) to detect different KEGG pathways between populations and between sexes (Parks et al., 2014). Two-sided Welch’s *t*-test method was utilized for the comparison of two groups. False discovery rate (FDR)-adjusted *p*-values were filtered at a significance level of 0.05.

To evaluate the effect of host genetic background on the gut microbial structure and function, we utilized the PASSaGE software (version 2.0.11.6) to perform Mantel tests on matrices (Rosenberg and Anderson, 2011), i.e., SSR-based Bray–Curtis distance, distances of ML and Bayesian trees, OTU-based distances (i.e., Bray–Curtis, Jaccard, weighted UniFrac, and unweighted UniFrac), and Bray–Curtis distances of KO and KEGG pathway tables. The R package “corrplot” (version 0.84) was taken to visualize these Mantel correlation values with FDR-adjusted *p* < 0.05.

To test whether life stage and health condition could produce a marked effect on the structure and function of gut microbiota, furthermore, we calculated Spearman correlations between life stage and RBT factors, α diversity indices, and relative abundances of taxonomic (i.e., sex- or population-biased taxa) and functional markers (i.e., sex or population-biased KEGG pathways) detected above. The R package “corrplot” was taken to visualize these Spearman correlation values with FDR-adjusted *p* < 0.05.

Subsequently, we utilized the R packages “vegan” and “plspm” (version 0.4.9) to evaluate the explanatory power of intrinsic factors for structural variations of gut microbiota. Specifically, redundancy analysis (RDA) and variation partitioning analysis (VPA) were performed on the rarefied OTU table with Hellinger transformation. Since two sample individuals (i.e., S12 and S36) possessed incomplete RBT factors (i.e., alkaline phosphatase unavailable in S12 and lactate dehydrogenase and creatine kinase unavailable in S36), we removed these two samples in RDA and VPA. The life stage factors except for body length and RBT factor of total protein were excluded in terms of variance inflation factors. After detecting significant constraint axes in RDA by the “anova.cca” function, we applied the “envfit” function to identify the life stage and RBT factors significantly fitting to these axes. The OTUs associated to the significant constraint axes (i.e., RDA1 and RDA2) in RDA were screened in terms of Spearman correlations with FDR-adjusted *p* < 0.05. We applied partial least squares path modeling (PLSPM) for life stage, location, and α diversity (or population-biased taxonomic compositions) to test whether life stage drove the structural divergence of gut microbiota between populations. As for location factors, we assigned “1” to “TJ” and “0” to “XZ.” The bootstrap method (number = 100) was utilized to evaluate the significance of direct path coefficients in PLSPM.

To test the effect of life stage on the topological properties of co-occurrence networks, we compared network parameters between younger (body length ≤ 8.4 cm, *n* = 10) and older (body length ≥ 9.8 cm, *n* = 11) samples. The co-occurrence networks were constructed using R package “igraph” in terms of Spearman correlations for 24 shared OTUs (existed in more than half of the samples in each group). Correlation coefficients were filtered at a minimum threshold of 0.6 with FDR-adjusted *p* < 0.05. Singleton nodes were removed in co-occurrence networks before calculation of network parameters. The keystone OTUs were predicted by using the criterion of both normalized degree and betweenness centrality > 0.1. To identify target OTUs of life stage in co-occurrence network remodeling, we also constructed a co-occurrence network for body length, Shannon index, and 37 shared OTUs in all samples.

## Results

### Gut Microbiota Structure and Function of Hibernating *Bufo gargarizans*

**FIGURE 1:**
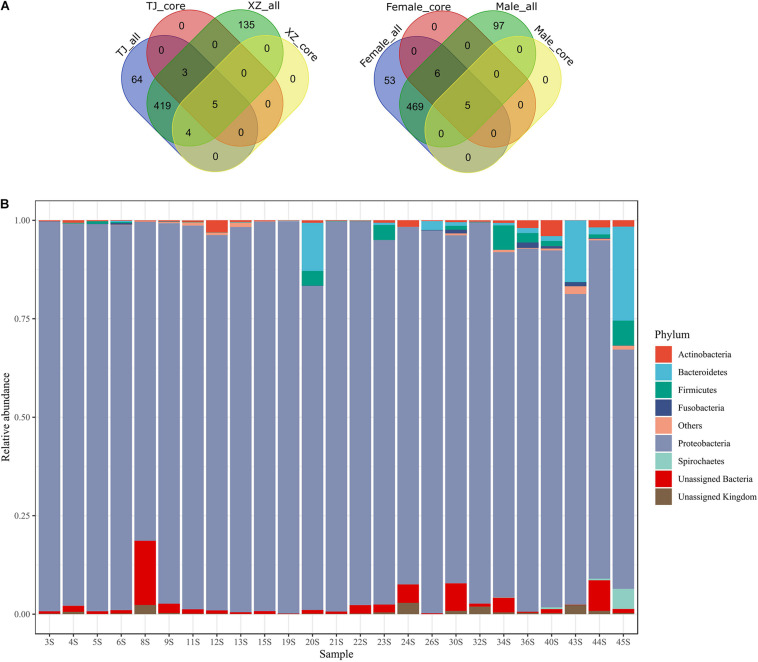
Distribution of OTUs between groups **(A)** and relative abundance of phyla in 25 gut microbiota samples **(B)**. In the Venn diagrams, each group (i.e., location or sex) with an “all” or “core” suffix represents total OTUs or core OTUs (i.e., 100% individual coverage) of a given group, respectively. The taxa with average relative abundance among individuals < 0.001 were grouped as “Others.”

We got 457,632 quality-controlled 16S rRNA gene amplicons in total (Supplementary Table 3). The length of these sequences ranged from 300 to 492 bases (mean = 423 bases). From these sequences, 630 OTUs were picked to annotate 412,647 sequences (i.e., 90.17% of total sequences) (Supplementary Material: raw_otu_table.txt and taxonomy_assignment.tsv). Specifically, the number of sequences matching OTUs ranged from 6,301 to 46,313 in 25 samples (mean = 16506). In total, we screened out an archaeal phylum (i.e., Crenarchaeota) and 18 bacterial phyla. The relative abundance of Proteobacteria (0.9196 ± 0.0892), which was the most dominant phylum in all samples, ranged from 60.78 to 99.60% (Figure 1). Five phyla, namely, Actinobacteria (0.0080 ± 0.0100), Bacteroidetes (0.0244 ± 0.0587), Firmicutes (0.0111 ± 0.0190), Fusobacteria (0.0019 ± 0.0038), and Spirochetes (0.0024 ± 0.0101), possessed a relative abundance more than 1% in at least one sample. The unassigned phylum taxa in gut microbiota occupied 0.16–18.64%. The top two dominant genera were assigned to be Pseudomonadaceae members, i.e., *Pseudomonas* (0.5023 ± 0.2314) and an unassigned genus (0.2662 ± 0.1147) (Supplementary Figure 3). The number of shared OTUs between populations and between sexes was 431 (68.41%) and 480 (76.19%), respectively (Figure 1). Neither location-specific (i.e., core OTUs unique for one location) nor sex-specific core OTUs (i.e., core OTUs unique for one sex) was detected. The counts of core OTUs in TJ and XZ populations were 8 and 9, but in male and female samples, these were 5 and 11 (Supplementary Table 4). The five core OTUs among all samples were classified into Proteobacteria comprising three families, i.e., Pseudomonadaceae (OTU1, OTU2, and OTU4), Enterobacteriaceae (OTU5), and Aeromonadaceae (OTU7). OTU1 (*Pseudomonas*), OTU2 (unassigned genus of Pseudomonadaceae), and OTU4 (*Pseudomonas*) were the three main members of the top two dominant genera among samples. Intriguingly, OTU2 was also classified (identity ≥ 0.97) as *Pseudomonas* by using SINA (version 1.2.11) in search of SILVA SSU Ref NR database (release 138.1^4^) (Pruesse et al., 2012). In addition, OTU5 and OTU7 without clear genus assignment based on Greengenes were classified by using the SINA tool as *Rahnella* (Yersiniaceae: a new family in Enterobacterales) and *Aeromonas*, respectively.

Due to method limitation, a lot of rare OTUs could not be successfully utilized in the PICRUSt analysis. Specifically, a small fraction of 630 OTUs (i.e., 30.63%) was identified as representative OTUs (based on 97% identity) of Greengenes (version 13.5). However, these dominant OTUs accounted for 83.61% of the quality-controlled sequences (i.e., 457,632). The top five predicted KEGG pathways were transporters (0.0634 ± 0.0046), ABC transporters (0.0409 ± 0.0026), general function prediction only (0.0375 ± 0.0012), two-component system (0.0319 ± 0.0018), and secretion system (0.0261 ± 0.0010) (Supplementary Figure 4).

### Greater Divergence of Gut Microbiota Between Populations Than Between Sexes

After rarefaction to a sampling depth of 6,301, 588 (93.33% of 630) OTUs were retained. The average OTU number of 25 samples was 98 with a standard deviation of 53 (Supplementary Table 5). We detected significant differences in Shannon (*p* < 0.001) and Pielou’s evenness (*p* < 0.001) but not in observed OTUs (*p* = 0.140) and Faith’s PD (*p* = 0.610) between two populations (Table 1). However, we did not detect any significant differences in α diversity indices between sexes. In addition, a significant interactive effect on Faith’s PD (*p* = 0.019) appeared to exist in sex and location factors. The location factor had significant effects on Bray–Curtis (*p* = 0.001) and Jaccard (*p* = 0.003) distance matrices of rarefied OTU table (Table 1), but no significant effects of sex were observed on these β diversity indices. Furthermore, significant differences in relative abundances were detected in 29 taxa between populations and three taxa between sexes (Supplementary Figure 5). After excluding higher-level taxa with equal relative abundances to their daughter taxa, we got 17 taxa (e.g., *Pseudomonas*) between two populations and one taxon (i.e., Chlamydiales) between two sexes. However, none of the KEGG pathways showed a significant difference between sexes or between populations. If unadjusted *p*-values were applied, a significant difference between two populations was detected in 81 KEGG pathways (Supplementary Figure 6). Thus, different populations owned greater divergence in gut microbiota than different sexes did.

**TABLE 1:** Two-way (sex and location) ANOVA and PERMANOVA on α and β diversity indices (i.e., Bray–Curtis and Jaccard distance matrices of rarefied OTU table, permutation number = 9,999).

	**Source of variation**	***df***	***F***	***p***
Observed OTUs	Sex	1	0.195	0.663
	Location	1	2.350	0.140
	Sex × location	1	3.879	0.062
Faith’s PD	Sex	1	0.055	0.817
	Location	1	0.268	0.610
	Sex × location	1	6.503	**0.019**
Shannon	Sex	1	0.375	0.546
	Location	1	14.611	**<0.001**
	Sex × location	1	0.084	0.775
Pielou’s evenness	Sex	1	0.326	0.574
	Location	1	19.359	**<0.001**
	Sex × location	1	0.892	0.356
Bray–Curtis distance	Sex	1	0.648	0.701
	Location	1	4.177	**0.001**
	Sex × location	1	2.056	0.070
Jaccard distance	Sex	1	0.722	0.661
	Location	1	3.548	**0.003**
	Sex × location	1	1.576	0.132

*The *p* value in bold means < 0.05.*

### No Detectable Host Genetic Effects on Structural and Functional Variation of Gut Microbiotas

The mtDNA and SSR markers generated an inconsistent genetic divergence pattern among these individuals [*r* = 0.095 (FDR-adjusted *p* = 0.309) and *r* = 0.090 (FDR-adjusted *p* = 0.309)] (Supplementary Figure 7, Supplementary Material: genetic_divergence.zip). In addition, neither of the host genetic divergence patterns possessed a significant correlation with structural and functional variation of gut microbiotas.

### Dominant Effects of Life Stage on the Gut Microbiota Discrepancy Between Populations

Life stage possessed significant Spearman correlations with two α diversity indices (i.e., Shannon and Pielou’s evenness), 16 population-biased taxa, and nine of top 10 (in terms of differences between relative abundances) potential population-biased KEGG pathways (Supplementary Figure 8). Specifically, morphological traits of life stage showed negative correlations with α diversity indices and XZ population-biased taxa and KEGG pathways but positive correlations with TJ population-biased ones. A few significant correlations were detected between population-biased taxa (and KEGG pathways) and five RBT factors (i.e., creatine kinase, aspartate aminotransferase, total protein, globulin, and alkaline phosphatase), though only two of them (i.e., creatine kinase and alkaline phosphatase) manifested a significant correlation with α diversity (Supplementary Figure 8). In addition, between-location difference was detected in creatine kinase and aspartate aminotransferase (Supplementary Tables 1, 6). No significant correlations were detected between RBT factors and life stage factors except creatine kinase (Supplementary Figure 8). Therefore, the gut microbiota discrepancy between populations might mainly result from the significant differences in life stage factors between populations.

**FIGURE 2:**
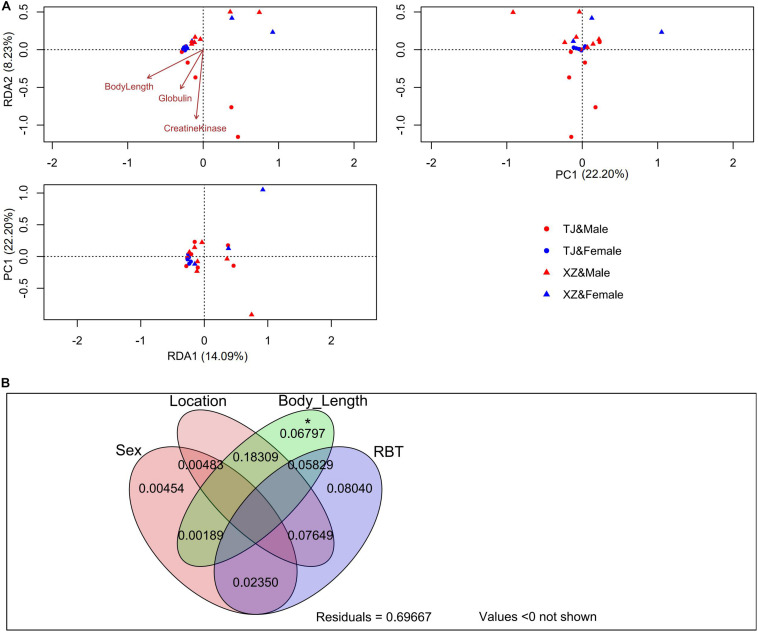
RDA **(A)** and VPA **(B)** on the rarefied OTU table with Hellinger transformation. The significance of each explained variation was tested by the permutation test under the reduced model (permutation number = 999). *p < 0.05.

In RDA analysis, we detected two significant constraint axes (i.e., RDA1 and RDA2) which interpreted 14.09 and 8.23% of gut microbiota variations (Figure 2). The TJ and XZ populations discriminated from each other in RDA1 and RDA2 dimensions. Body length, globulin, and creatine kinase were significantly associated with the gut microbiota variation based on RDA1 and RDA2. We identified three (i.e., OTU1, OTU3, and OTU4) and two OTUs (i.e., OTU6 and OTU68) significantly correlated with RDA1 and RDA2, respectively. However, the gut microbiota variation explained by constrained axes (i.e., 0.302) was much less than that explained by unconstrained axes. This phenomenon was confirmed by VPA results that sex, location, body length, and RBT accounted for 30.33% of gut microbiota variation (Figure 2). In addition to the dominant effect of body length on the variation associated with location, body length was the only one factor to singly explain the variation significantly.

**FIGURE 3:**
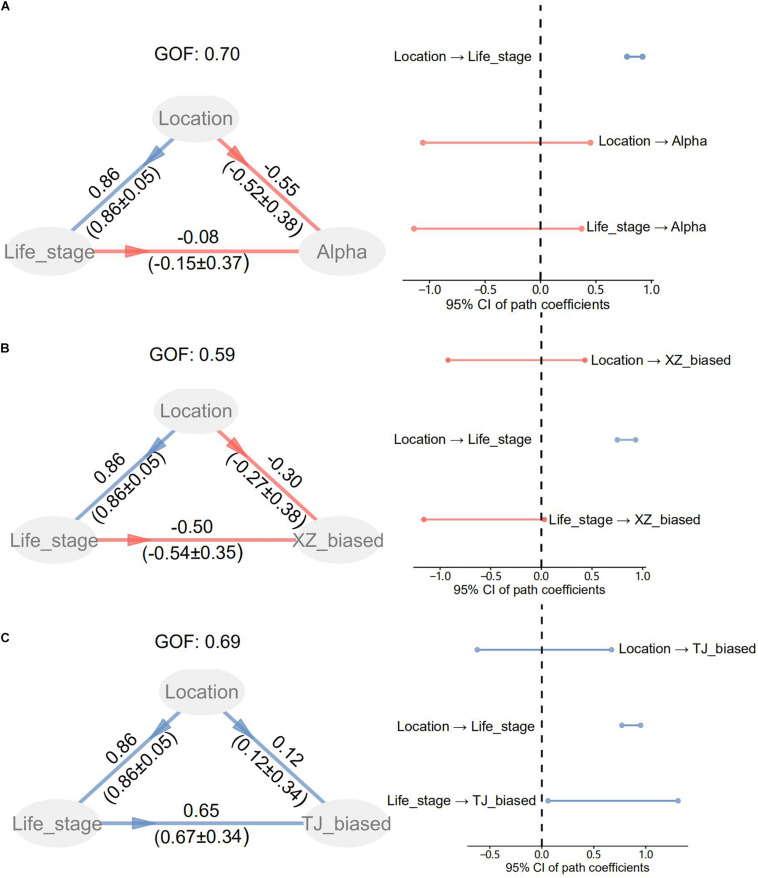
Partial least squares path modeling (PLSPM) for life stage, location, and α diversity **(A)** or population-biased taxonomic compositions **(B,C)**. As for location factors, “1” was assigned to TJ and “0” to XZ. TJ_biased and XZ_biased represent taxa with a biased relative abundance to TJ and XZ, respectively. GOF is the goodness of fit index for accounting for the model quality. The original path coefficients and bootstrap values (mean±SE) are shown along paths.

The PLSPM analyses confirmed the significant difference in life stage between two locations (Figure 3). No significant direct path coefficients were detected from location and life stage to α diversity indices. However, a significant direct coefficient probably existed in paths from life stage to compositions of location-biased taxa.

### Discrepant Co-occurrence Networks of Gut Microbiota Between Younger and Older Toads

**FIGURE 4:**
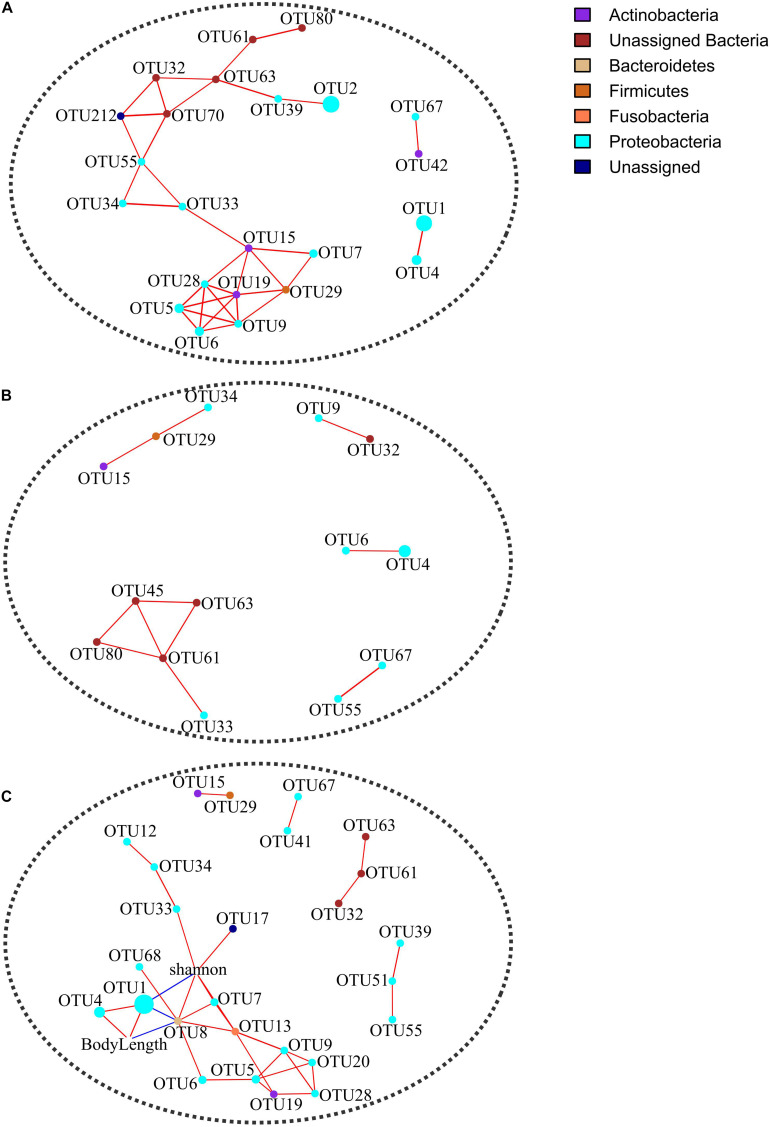
Co-occurrence networks in terms of Spearman correlations for 24 shared OTUs in younger **(A)** and older **(B)** groups and for 37 shared OTUs in all samples **(C)**. Correlation coefficients were filtered at a minimum threshold of 0.6 with FDR-adjusted *p* < 0.05. Singleton nodes were removed. Node size is positively correlated with the relative abundance of an OTU.

The OTU co-occurrence networks were significantly different between younger and older groups (Figure 4 and Table 2). Specifically, six network parameters (i.e., edge count, node count, diameter, density, average path length, and normalized betweenness centrality) of the younger group were greater than those of the older group. Five keystone OTUs (i.e., OTU15, OTU33, OTU55, OTU63, and OTU70) were identified in the younger group but none in the older group. From the co-occurrence network of all samples (Figure 4), one keystone OTU (i.e., OTU8) was identified. In addition, we identified three target OTUs (i.e., OTU1, OTU4, and OTU8) associated with life stage (i.e., body length) in co-occurrence network remodeling.

**TABLE 2:** Topological properties of the OTU co-occurrence networks in younger (body length ≤ 8.4 cm, *n* = 10) and older (body length ≥ 9.8 cm, *n* = 11) groups of hibernating *B. gargarizans*.

Parameters	Younger	Older	*p*
Edge count	34	11	–
Node count	23	14	–
Diameter	8	2	–
Density	0.134	0.121	–
Average path length	3.590	1.312	–
Normalized degree centrality (mean ± SD)	0.134 ± 0.072	0.121 ± 0.072	0.475
Normalized betweenness centrality (mean ± SD)	0.084 ± 0.128	0.004 ± 0.012	**0.016**

**p* shows the significance level of Mann–Whitney test on parameters between the younger and older groups. The *p* value in bold means < 0.05.*

## Discussion

Here, we explored the small intestine microbiota of hibernating Asiatic toads (*B. gargarizans*) in two wild populations. Similar to the large or whole intestine microbiota in hibernating amphibians (e.g., *Rana amurensis*, *R. dybowskii*, *R. sylvatica*, and *Polypedates megacephalus*) (Weng et al., 2016; Wiebler et al., 2018; Tong et al., 2019a, 2020), Proteobacteria, Bacteroidetes, and Firmicutes dominate in the small intestine microbiota of hibernating *B. gargarizans* (Figure 1). However, the relative abundances of these three phyla in this study are dramatically different from those in previous studies on the large or whole intestine microbiota. For instance, Proteobacteria inhabits small intestine microbiota with the highest proportion (0.9196 ± 0.0892). In comparison with active individuals, the relative abundance of Proteobacteria tends to be higher in the large or whole gut microbiota of hibernators (Weng et al., 2016; Tong et al., 2020), which is not applicable for all amphibians (Wiebler et al., 2018). Given the comparable relative abundance of Proteobacteria in the small and large intestine microbiota of active amphibians (Zhang et al., 2018; Zhou et al., 2020), the increase rate of Proteobacteria in the small intestine seems to be greater than that in the large intestine during hibernation. Since we have no data on the small gut microbiota of non-hibernating toads, nevertheless, and the hypothesis remains untested.

The depression of small intestinal performance with fasting (e.g., decreases in intestinal mass and length, enterocyte size, mucosal thickness, and digestive capacities) is evolutionarily prevailing in amphibians and more severe during estivation or hibernation (Cramp et al., 2005; Secor, 2005; Naya et al., 2009; Tamaoki et al., 2016; Wiebler et al., 2018). The dietary shift from external nutrition to intestinal mucus during hibernation is probably a key determinant factor in reshaping the gut microbiota of amphibians (e.g., reduction of bacterial number and increase of the Bacteroidetes/Firmicutes ratio) (Weng et al., 2016; Wiebler et al., 2018; Tong et al., 2020), though no data are about the small intestine microbiota. *Pseudomonas* is a facultative Gram-negative bacterial genus ubiquitously distributed in nature (e.g., drinking water) and host-associated environments (e.g., intestines) (Vaz-Moreira et al., 2017; de la Maza et al., 2020b). Although *Pseudomonas* spp. are often treated as opportunistic pathogens for amphibians (Weng et al., 2016; Tong et al., 2019b), moderate accumulation of *Pseudomonas* in the large intestine appears to be a trade-off between cold adaptation (e.g., urea–nitrogen recycling) and immune costs (Wiebler et al., 2018). Intriguingly, we detected high accumulation of *Pseudomonas* in the small intestine of hibernating Asiatic toads (Supplementary Figure 3). One candidate reason for the phenomenon is more appropriate conditions (e.g., higher oxygen or easier accessibility for external *Pseudomonas*) in small intestines than large intestines (Martinez-Guryn et al., 2019). Moreover, the predominant KEGG pathways predicted (e.g., ABC transporters) tend to be involved in environmental information processing (Supplementary Figure 4). According to significant correlations between *Pseudomonas* and the dominant KEGG pathways (e.g., two-component system), *Pseudomonas* appears to be a key adaptor for small gut microbiota during hibernation (Supplementary Figure 8). Here, we also identified two subdominant core OTUs (assigned as *Rahnella* and *Aeromonas*) widely distributed in aqueous environments (Kämpfer, 2015; de la Maza et al., 2020a), which could be related with the subaqueous hibernation style of Asiatic toads. In addition, the co-occurrence of psychrotolerant *Rahnella* and pathogenic *Aeromonas* appears to coincide with the trade-off hypothesis between cold adaptation and immune cost.

To effectively manipulate the gut microbiota and maintain the host health, we need to illuminate the association between driving factors and gut microbiota. However, it is a big challenge in the case that multiple driving factors play synergistic effects on gut microbiota. For instance, the gut microbiota discrepancy between geographical populations has been reported in many amphibian species, e.g., *B. gargarizans*, *Fejervarya limnocharis*, *Babina adenopleura*, and *Salamandra salamandra* (Bletz et al., 2016; Vences et al., 2016; Huang et al., 2017; Xu et al., 2020). In most cases, the interpopulation gut microbiota discrepancy can be attributed to the divergence of habitats (e.g., dietary structures and bacterial species pools) rather than genetic backgrounds. Even the species effect on the gut microbiota sometimes is subdominant to that of habitats. For instance, the habitat factor rather than the species factor explained significant structural and functional variation in gut microbiota of *F. limnocharis* and *B. adenopleura* (Huang et al., 2017). Due to a long-term fasting, hibernating amphibians provide an ideal experimental platform to evaluate the effects of non-dietary factors on the gut microbiota. For example, an interspecific divergence was detected in the gut microbiota of two sympatric frogs (i.e., *R. amurensis* and *R. dybowskii*) during hibernation (Tong et al., 2019a).

The present study detected a significant discrepancy of small intestine microbiota between two geographical populations of hibernating Asiatic toads. However, the between-population discrepancy appears not to be decided by population-specific habitats. First, habitats during hibernation tend to be homogeneous between populations, such as long-term fasting and subaqueous hibernation. Second, Tianjian (Ji Canal) and Xuzhou (Luoma Lake) populations are located in similar ecological surroundings affected by human activities. Third, no location-specific core OTUs (i.e., 100% sample occurrence in a population) were identified for the two populations. Due to a geographical distance of about 600 km between two locations (Supplementary Figure 1), nevertheless, incongruent hibernation span in the two populations could be true and contributed to the gut microbiota discrepancy between populations (Fei et al., 2012). In addition, microhabitats of Asiatic toads during the active period might be heterogeneous in dietary structures and bacterial species pools. Location-specific OTUs accounting for 31.59% of total ones suggest a potential divergence of bacterial species pools between populations (Figure 1). However, the sample occurrence of these location-specific OTUs depicts a similar skewed distribution (e.g., 39.20% existence in a single individual) as that of location-shared OTUs. The phenomenon indicates that a high contingency of gut bacterial species exists among individuals and the contingency is similar between location-specific and location-shared OTUs. Since Asiatic toads (even in the same population) have a broad-spectrum diet (e.g., insects and earthworms) (Yu et al., 2009), the high heterogeneity in diet may contribute to the high contingency. Therefore, habitat (e.g., bacterial species pool) appears to play a weak role in the gut microbiota discrepancy between these two populations of hibernating Asiatic toads.

Although sex-specific effects may occur in the ecological adaptation of gut microbiota (Ling et al., 2020), here we did not detect significant between-sex differences in terms of α and β diversity indices (Table 1), except for a female-biased taxon, i.e., pathogenic Chlamydiales (Borel et al., 2018). The VPA result indicates that sex is not an important driving factor for the gut microbiota variation among hibernating Asiatic toads (*R*^2^ = 0.003, *p* = 0.344). As for the significant interactive effect on Faith’s PD (*p* = 0.019) between sex and location factors, a candidate reason is a by-product from the sampling bias on life stage (Supplementary Table 6). Specifically, life stage factors showed a significant between-location difference. In addition, life stage factors (e.g., body length) also manifested a significant interactive effect between sex and location factors. Anyway, it is just a bold deduction based on the driving effect of life stage on the gut microbiota (see following discussion in detail). The insignificant co-variation between genetic divergence patterns and β diversity of gut microbiota (Supplementary Figure 7) suggests that host genetic background may possess a minor explanatory capacity on the gut microbial variation in hibernating Asiatic toads. Although we utilized two types of markers (nuclear and mitochondrial DNA) to deduce genetic divergence patterns, the kinship and relatedness could be biased. For instance, the inconsistent genetic divergence patterns probably result from the different inheritance rules for nuclear (biparental transmission) and mitochondrial DNA (maternal transmission). To identify the genetic materials filtering gut microbiota of Asiatic toads at the population scale, it is necessary to carry out an in-depth investigation from the genomic level (Lu et al., 2021). The life stage factors of the TJ population were significantly larger than those of the XZ population in this study (Supplementary Tables 1, 6). Although these factors (e.g., body length) cannot give the exact life stage (i.e., age) of Asiatic toads due to latitude- and sex-dependent variation (Yu and Lu, 2013), these two populations sampled are evidently different in life stage. In addition, the life stage factors possessed significant correlations with population-biased taxa and KEGG pathways (Supplementary Figure 8). The life stage has been identified an important determinant for the gut microbiota assembly of amphibians (Kohl et al., 2013; Knutie et al., 2017; Warne et al., 2017; Chai et al., 2018; Zhang et al., 2018). Therefore, it is reasonable to hypothesize that the sampling bias on life stage caused the between-population discrepancy in the gut microbiota. Even though the RBT factors also showed a certain degree of association with population-biased taxa and KEGG pathways, the association might be a by-product generated by the effects of life stage on the gut microbiota. For instance, creatine kinases play an essential role in creatine/phosphocreatine shuttle system to assist ATP hydrolysis and can be applied as an indicator of muscle degradation (Sumien et al., 2018). A positive correlation between creatine kinase and life stage suggests that older toads may possess greater muscle degradation than younger toads during hibernation. Nevertheless, we cannot exclude the possibility that some life stage-irrelevant RBT factors (e.g., aspartate aminotransferase) are able to explain a fraction of gut microbial variation, e.g., through blood metabolites (Wilmanski et al., 2019). The above hypothesis was supported by the subsequent analyses including RDA, VPA, and PLSPM (Figures 2, 3). Although body length and two RBT factors (i.e., creatine kinase and globulin) are significantly associated with the gut microbiota variation, the dominant factor for the gut microbiota variation is body length (*R*^2^ = 0.128, *p* = 0.001) rather than RBT factors (*R*^2^ = 0.056, *p* = 0.255). In addition, the VPA result also revealed that the fraction of variation explained by location (*R*^2^ = 0.081, *p* = 0.005) is closely related with body length (*R*^2^ = 0.183). The PLSPM analyses further confirmed that life stage (i.e., body length) is the decisive factor for the gut microbiota discrepancy between two populations (Figure 3). In accordance with a previous study on pig gut microbiome (Ke et al., 2019), a life stage-linked discrepancy of OTU co-occurrence networks was detected in the hibernating Asiatic toads (Figure 4). The microbial network of the older group tends to be simpler than that of the younger group during hibernation. *Pseudomonas* (i.e., OTU1 and OTU4) and *Bacteroides* (i.e., OTU8) were identified as the effect targets of life stage in the co-occurrence network analysis. Intriguingly, we also screened out *Pseudomonas* (i.e., OTU1 and OTU4), *Serratia* (i.e., OTU3), *Citrobacter* (i.e., OTU6), and *Shewanella* (i.e., OTU68) associated with the first two constraint axes in RDA. These opportunistic pathogens and/or cold-stress-related bacteria (e.g., *Pseudomonas* and *Citrobacter*) are significantly correlated with life stage (Supplementary Figure 8; Wiebler et al., 2018), suggesting that the responses to hibernation appear to be distinct in the gut microbiota across different adult stages.

To sum, the small intestine microbiota of adult hibernating Asiatic toads from two wild populations is characterized by a highest relative abundance of Proteobacteria, especially *Pseudomonas*. Sex and genetic background tend to play a minor role in the gut bacterial community of hibernating Asiatic toads. The significant discrepancy of gut microbiota between geographical locations mainly results from the life stage. However, most of the gut microbiota variation (∼70%) in the two wild populations of hibernating Asiatic toads cannot be explained by the deterministic factors measured in this study, such as life stage. According to the significantly skewed distribution of location-specific OTUs, the gut microbiota of hibernating Asiatic toads seems to be driven by not only deterministic processes but also stochastic processes, e.g., historical contingency (Zhou and Ning, 2017). Finally, limitations do exist in our study based on wild populations of hibernating Asiatic toads, such as uncontrolled historical contingency in diets and habitats. Therefore, further studies on gut microbiota of hibernating amphibians need improvements with larger sample size and/or more precise control of untargeted factors.

## Data Availability Statement

The datasets presented in this study can be found in online repositories. The names of the repository/repositories and accession number(s) can be found below: https://db.cngb.org/search/project/CNP0001473/ and https://db.cngb.org/search/project/CNP0001644/.

## Ethics Statement

The animal study was reviewed and approved by Animal Care and Use Committee of Xinyang Normal University.

## Author Contributions

XS designed the study, analyzed the data, and wrote the manuscript. XS, JZ, JS, and YZ performed the experiments and collected the data. All authors read and approved the final manuscript.

## Conflict of Interest

The authors declare that the research was conducted in the absence of any commercial or financial relationships that could be construed as a potential conflict of interest.

## Publisher’s Note

All claims expressed in this article are solely those of the authors and do not necessarily represent those of their affiliated organizations, or those of the publisher, the editors and the reviewers. Any product that may be evaluated in this article, or claim that may be made by its manufacturer, is not guaranteed or endorsed by the publisher.

## Footnotes

1
http://bioinformatics.psb.ugent.be/webtools/Venn/

2
http://galaxy.morganlangille.com/

3
http://huttenhower.sph.harvard.edu/galaxy/

4
https://www.arb-silva.de/
