# Supplementary material for: Decisive Effects of Life Stage on the Gut Microbiota Discrepancy Between Two Wild Populations of Hibernating Asiatic Toads (Bufo gargarizans)
Source: Front Microbiol. 2021 Aug 3;12:665849. doi: 10.3389/fmicb.2021.665849 (PMC8369469; doi:10.3389/fmicb.2021.665849)
Supplement: Supplementary file 1 [file Data_Sheet_1.zip › Supplementary Material/Supplementary Tables and Figure Captions.docx]

**Supplementary Table 1. Summary for intrinsic factors of 25 samples in two populations. Abbreviates: TJ (Tianjin), XZ (Xuzhou), F (female), M (male).**

| **Sample ID** | **Location** | **Sex** | **Body Length (cm)** | **Body Mass (g)** | **BM:BL (g/cm)** | **Eye Space (cm)** | **Nasal Space (cm)** | **Lactate Dehydrogenase (×20 U/L)** | **Creatine Kinase (×20 U/L)** | **Alanine Aminotransferase (U/L)** | **Aspartate Aminotransferase (U/L)** | **Total Protein (g/L)** | **Albumin (g/L)** | **Globulin (g/L)** | **Alkaline Phosphatase (U/L)** |
| --- | --- | --- | --- | --- | --- | --- | --- | --- | --- | --- | --- | --- | --- | --- | --- |
| S11 | TJ | F | 10.9 | 173.76 | 15.94 | 2.3 | 0.7 | 109 | 312 | 3 | 465 | 23.5 | 13.9 | 9.6 | 10 |
| S12 | TJ | M | 10.5 | 140.05 | 13.34 | 2 | 0.75 | 167 | 320 | 21 | 937 | 30.5 | 17.5 | 13 | n.a. |
| S13 | TJ | F | 11 | 177.37 | 16.12 | 2.1 | 0.7 | 93 | 182 | 5 | 592 | 26.7 | 17.1 | 9.6 | 8 |
| S15 | TJ | M | 9.1 | 87.99 | 9.67 | 2.1 | 0.6 | 158 | 361 | 48 | 0 | 35.8 | 17.4 | 18.4 | 15 |
| S19 | TJ | M | 8.9 | 98.89 | 11.11 | 2 | 0.6 | 272 | 987 | 10 | 689 | 34.6 | 14.9 | 19.7 | 13 |
| S20 | TJ | M | 9.8 | 117.2 | 11.96 | 2.3 | 0.7 | 127 | 358 | 14 | 0 | 35 | 18.8 | 16.2 | 0 |
| S21 | TJ | M | 9 | 92.79 | 10.31 | 2 | 0.6 | 127 | 227 | 12 | 569 | 36 | 17.2 | 18.8 | 12 |
| S22 | TJ | F | 11.4 | 188.12 | 16.5 | 2.3 | 0.8 | 75 | 190 | 10 | 522 | 30.6 | 15.9 | 14.7 | 18 |
| S23 | XZ | M | 7.9 | 55.73 | 7.05 | 1.8 | 0.5 | 217 | 234 | 23 | 910 | 17.5 | 12.6 | 4.9 | 4 |
| S24 | XZ | M | 8.4 | 75.44 | 8.98 | 1.8 | 0.6 | 267 | 268 | 18 | 947 | 32.2 | 20 | 12.2 | 14 |
| S26 | XZ | M | 7.8 | 58.1 | 7.45 | 1.8 | 0.6 | 264 | 300 | 20 | 953 | 33 | 19.2 | 13.8 | 25 |
| S30 | XZ | M | 9 | 91.8 | 10.2 | 1.8 | 0.6 | 148 | 268 | 10 | 888 | 31.7 | 21.1 | 10.6 | 4 |
| S32 | XZ | F | 8.2 | 73.99 | 9.02 | 1.8 | 0.6 | 165 | 184 | 15 | 937 | 25.6 | 15.1 | 10.5 | 6 |
| S34 | XZ | M | 7.5 | 64.22 | 8.56 | 1.5 | 0.5 | 382 | 310 | 9 | 816 | 32.7 | 19.2 | 13.5 | 0 |
| S36 | XZ | M | 7.4 | 45.35 | 6.13 | 1.5 | 0.5 | n.a. | n.a. | 14 | 1041 | 25.7 | 19.1 | 6.6 | 0 |
| S3 | TJ | M | 10.5 | 171.46 | 16.33 | 2.3 | 0.7 | 204 | 425 | 88 | 201 | 29.2 | 17.4 | 11.8 | 11 |
| S40 | XZ | F | 7.2 | 45.18 | 6.28 | 1.6 | 0.5 | 133 | 53 | 28 | 636 | 47.3 | 24.6 | 22.7 | 0 |
| S43 | XZ | F | 6.4 | 33.63 | 5.25 | 1.5 | 0.5 | 133 | 171 | 11 | 644 | 7.7 | 9.1 | 0 | 0 |
| S44 | XZ | M | 7 | 34.6 | 4.94 | 1.4 | 0.5 | 134 | 51 | 8 | 589 | 25.4 | 17 | 8.4 | 28 |
| S45 | XZ | M | 6.45 | 30.13 | 4.67 | 1.3 | 0.5 | 91 | 59 | 5 | 678 | 15.5 | 13.9 | 1.6 | 0 |
| S4 | TJ | F | 11.1 | 180 | 16.22 | 2.3 | 0.8 | 86 | 220 | 10 | 708 | 27.8 | 15.8 | 12 | 8 |
| S5 | TJ | M | 11.3 | 195.5 | 17.3 | 2.4 | 0.8 | 159 | 440 | 10 | 981 | 34.9 | 19 | 15.9 | 11 |
| S6 | TJ | M | 11.1 | 156.5 | 14.1 | 2.3 | 0.7 | 113 | 334 | 10 | 695 | 36.2 | 18.1 | 18.1 | 18 |
| S8 | TJ | F | 11.4 | 190.7 | 16.73 | 2.4 | 0.7 | 178 | 477 | 10 | 766 | 23.8 | 13.3 | 10.5 | 4 |
| S9 | TJ | F | 11 | 181.23 | 16.48 | 2.3 | 0.8 | 70 | 188 | 3 | 530 | 15.2 | 9.9 | 5.3 | 8 |

**Supplementary Table 2. Primers for mtDNA (D-loop and cytb) and SSR markers.**

| **Gene loci** | **Primer 1 (5’→3’)** | **Primer 2 (5’→3’)** | **References** |
| --- | --- | --- | --- |
| D-loop | Bu-con-15971F: GAGCCTTCCCTTGGTTTAAGAGTA | Bu-con-16582R: CCAGGTTAAGGTCTTTAAGGTACCAG | ([1](#_ENREF_1)) |
| cytb | Bg12-2L: ATTRTCTTACAGCTCGTAAG | Bg12-H: GCTACTAGTGTCCAGAAGAA | ([2](#_ENREF_2)) |
| Bbufu11 | GTCACATGGATAATAAATGAGACC | TCTAATATTGATGACCAGACAACC | ([3](#_ENREF_3)) |
| Bbufu14 | CGTGCATGCAAGTGTACCTAACC | ATGGAGAGTGAAGGGGAAAGAGTG | ([3](#_ENREF_3)) |
| Bbufu23 | ATCGCGGTGGCTGATGG | TGTGTATAATTTTGCCCGTTTAGG | ([3](#_ENREF_3)) |
| ATM-7 | ATGCTAATCCGCAAAGCGTA | CCACTGTACACGCCTCACAA | ([4](#_ENREF_4)) |
| ATM17 | GGAGGACTTGGAGAGGATGG | GATCCCTGCACCACCAAATA | ([4](#_ENREF_4)) |
| ATM18 | GGCAGGAGACACCTGACTGT | ATGGCCGATGTGACAAGTCT | ([4](#_ENREF_4)) |
| ATM27 | AGAGGGAGTCCGAGGATGAC | TCACCGACTGACTCCTGGAT | ([4](#_ENREF_4)) |
| ATM35 | CATGGCCTGCAGTATCAGTG | CATTACTGCTGCTCCCTGCT | ([4](#_ENREF_4)) |

1. Yu, T. L., Lin, H. D., and Weng, C. F. (2014) A new phylogeographic pattern of endemic *Bufo bankorensis* in Taiwan Island is attributed to the genetic variation of populations. *PloS one* **9**, e98029

2. Cao, S. Y., Wu, X. B., Yan, P., Hu, Y. L., Su, X., and Jiang, Z. G. (2006) Complete nucleotide sequences and gene organization of mitochondrial genome of *Bufo gargarizans*. *Mitochondrion* **6**, 186-193

3. Brede, E. G., Rowe, G., Trojanowski, J., and Beebee, T. J. C. (2001) Polymerase chain reaction primers for microsatellite loci in the Common Toad *Bufo bufo*. *Molecular Ecology Notes* **1**, 308-310

4. Li, N., Wen, G., Yang, W., and Fu, J. (2015) Isolation and characterization of fourteen microsatellite loci for Asiatic toad (*Bufo gargarizans*) at high altitude through transcriptome sequencing. *Conservation Genetics Resources* **7**, 407-409

**Supplementary Table 3. Summary for quality-controlled sequences and OTUs in 25 samples.**

| **Items** | **Values** |
| --- | --- |
| Number of total sequences | 457632 |
| Number of total bases | 193535186 |
| Minimum sequence length | 300 |
| Maximum sequence length | 492 |
| Mean of sequence lengths | 423 |
| Number of OTUs | 630 |
| Number of total sequences matching OTUs | 412647 (90.17%) |

**Supplementary Table 4. Core OTUs in different groups and taxonomic annotations based on Greengenes (ver. 13.8). The taxonomic names with * represent that those OTUs without clear taxonomic assignment based on Greengenes (ver. 13.8) were classified (identity ≥ 0.97) by using SINA (ver. 1.2.11) in search of SILVA SSU Ref NR database (release 138.1, https://www.arb-silva.de/). The relative abundance of core OTUs across all sample individuals (*n* = 25) is shown as mean±SD.**

| **Core OTU (Group)** | **Phylum** | **Family** | **Genus** | **Relative abundance** |
| --- | --- | --- | --- | --- |
| OTU1 (All) | Proteobacteria | Pseudomonadaceae | *Pseudomonas* | 0.3925±0.1752 |
| OTU2 (All) | Proteobacteria | Pseudomonadaceae | *Pseudomonas** | 0.2662±0.1147 |
| OTU4 (All) | Proteobacteria | Pseudomonadaceae | *Pseudomonas* | 0.1085±0.0583 |
| OTU5 (All) | Proteobacteria | Enterobacteriaceae/ Yersiniaceae* | *Rahnella** | 0.0262±0.0802 |
| OTU6 (Female/XZ) | Proteobacteria | Enterobacteriaceae | *Citrobacter* | 0.0211±0.0557 |
| OTU7 (All) | Proteobacteria | Aeromonadaceae | *Aeromonas** | 0.0132±0.0314 |
| OTU15 (Female/XZ) | Actinobacteria | Microbacteriaceae | *Clavibacter* | 0.0015±0.0035 |
| OTU32 (Female/XZ) | Unassigned Bacteria | — | — | 0.0008±0.0008 |
| OTU33 (Female) | Proteobacteria | Enterobacteriaceae | *Escherichia-Shigella** | 0.0015±0.0027 |
| OTU61 (Female/TJ) | Unassigned Bacteria | — | — | 0.0008±0.0007 |
| OTU67 (Female/XZ) | Proteobacteria | Moraxellaceae | *Acinetobacter* | 0.0004±0.0007 |
| OTU80 (TJ) | Unassigned Bacteria | — | — | 0.0003±0.0002 |
| OTU156 (TJ) | Unassigned Bacteria | — | — | 0.0002±0.0002 |

**Supplementary Table 5. The values of α diversity index among 25 samples at the rarefication depth of 6301.**

| **Sample ID** | **Pielou’s evenness** | **Faith’s PD** | **observed OTUs** | **Shannon** |
| --- | --- | --- | --- | --- |
| S11 | 0.2936 | 22.3323 | 103 | 1.9634 |
| S12 | 0.3266 | 15.4213 | 76 | 2.0408 |
| S13 | 0.3106 | 15.3787 | 74 | 1.9284 |
| S15 | 0.2823 | 15.4465 | 53 | 1.6172 |
| S19 | 0.2933 | 7.0863 | 30 | 1.4390 |
| S20 | 0.4311 | 16.5117 | 72 | 2.6602 |
| S21 | 0.3046 | 17.0350 | 70 | 1.8667 |
| S22 | 0.2689 | 33.2644 | 96 | 1.7704 |
| S23 | 0.4009 | 26.0861 | 90 | 2.6028 |
| S24 | 0.3215 | 36.8814 | 103 | 2.1497 |
| S26 | 0.3414 | 9.4799 | 36 | 1.7648 |
| S30 | 0.4474 | 61.5112 | 180 | 3.3520 |
| S32 | 0.3394 | 15.6440 | 86 | 2.1813 |
| S34 | 0.4169 | 52.9407 | 170 | 3.0891 |
| S36 | 0.5147 | 14.9275 | 109 | 3.4833 |
| S3 | 0.2774 | 14.2917 | 52 | 1.5816 |
| S40 | 0.4694 | 22.0197 | 158 | 3.4284 |
| S43 | 0.5563 | 12.0139 | 54 | 3.2016 |
| S44 | 0.4652 | 66.3006 | 244 | 3.6893 |
| S45 | 0.4053 | 27.2332 | 110 | 2.7484 |
| S4 | 0.2814 | 18.1888 | 96 | 1.8529 |
| S5 | 0.3192 | 12.0316 | 43 | 1.7321 |
| S6 | 0.3323 | 18.0725 | 79 | 2.0950 |
| S8 | 0.4111 | 77.0291 | 192 | 3.1183 |
| S9 | 0.2991 | 22.3644 | 88 | 1.9318 |

**Supplementary Table 6. Two-way (sex and location) ANOVA on intrinsic factors.**

|  | Source of variation | df | F | p |
| --- | --- | --- | --- | --- |
| Body Length | sex | 1 | 1.059 | 0.315 |
|  | location | 1 | 84.870 | **<0.001** |
|  | sex×location | 1 | 5.103 | **0.035** |
| Body Mass | sex | 1 | 3.486 | 0.076 |
|  | location | 1 | 79.229 | **<0.001** |
|  | sex×location | 1 | 5.680 | **0.027** |
| BM:BL | sex | 1 | 2.692 | 0.116 |
|  | location | 1 | 73.460 | **<0.001** |
|  | sex×location | 1 | 4.358 | **0.049** |
| Eye Space | sex | 1 | 0.780 | 0.387 |
|  | location | 1 | 68.751 | **<0.001** |
|  | sex×location | 1 | 0.358 | 0.556 |
| Nasal Space | sex | 1 | 1.458 | 0.241 |
|  | location | 1 | 45.406 | **<0.001** |
|  | sex×location | 1 | 1.858 | 0.187 |
| Lactate Dehydrogenase | sex | 1 | 5.485 | **0.030** |
|  | location | 1 | 2.471 | 0.132 |
|  | sex×location | 1 | 0.015 | 0.904 |
| Creatine Kinase | sex | 1 | 2.971 | 0.100 |
|  | location | 1 | 5.774 | **0.026** |
|  | sex×location | 1 | 0.423 | 0.523 |
| Alanine Aminotransferase | sex | 1 | 1.080 | 0.311 |
|  | location | 1 | 0.020 | 0.888 |
|  | sex×location | 1 | 2.798 | 0.109 |
| Aspartate Aminotransferase | sex | 1 | 0.013 | 0.909 |
|  | location | 1 | 4.794 | **0.040** |
|  | sex×location | 1 | 0.829 | 0.373 |
| Total Protein | sex | 1 | 1.822 | 0.191 |
|  | location | 1 | 0.540 | 0.471 |
|  | sex×location | 1 | 1.946 | 0.178 |
| Albumin | sex | 1 | 2.700 | 0.115 |
|  | location | 1 | 0.574 | 0.457 |
|  | sex×location | 1 | 0.361 | 0.554 |
| Globulin | sex | 1 | 0.942 | 0.343 |
|  | location | 1 | 2.572 | 0.124 |
|  | sex×location | 1 | 3.903 | 0.061 |
| Alkaline Phosphatase | sex | 1 | 1.858 | 0.188 |
|  | location | 1 | 1.826 | 0.192 |
|  | sex×location | 1 | 0.578 | 0.456 |

**Supplementary Figure 1. The geographical locations of two wild populations of hibernating Asiatic toads (*Bufo gargarizans*) and monthly average temperature (MAT) in 2014 of the locations. The MAT data in 2014 for two locations (Tianjin [54527], Xuzhou [58027]) were acquired from China Meteorological Data Service Center (http://data.cma.cn/). XZ, Xuzhou; TJ, Tianjin.**

**Supplementary Figure 2. Spearman correlation coefficients of α or β diversity indices between 10 iterative rarefied OTU tables at a sampling depth of 6000 or 6301.**

**Supplementary Figure 3. Relative abundance of genera in 25 gut microbiota samples. The taxa with average relative abundance among individuals <0.01 were grouped as “Others”.**

**Supplementary Figure 4. Relative abundance of top nine KEGG pathways in terms of average relative abundance among 25 gut microbiota samples.**

**Supplementary Figure 5. Identification of taxonomic markers for gut microbiota between in two populations (a) and sexes (b) using the LEfSe method (online Galaxy version 1.0, http://huttenhower.sph.harvard.edu/galaxy/). Per-sample sum was normalized to one million as the algorithm designers recommend. Both alpha values for the factorial Kruskal-Wallis test among classes and the pairwise Wilcoxon test between subclasses were set to 0.01. The threshold on the logarithmic LDA score for discriminative features was set to 2.0. All-against-all strategy was executed for multi-class analysis.**

**Supplementary Figure 6. Different KEGG pathways between populations detected using the STAMP software (ver. 2.1.3) based on unadjusted *P* values. Two-sided Welch’s t-test method was utilized.**

**Supplementary Figure 7. Mantel tests on matrices, i.e., SSR-based Bray-Curtis distance, distances of ML and Bayesian trees, OTU-based distances (i.e., Bray-Curtis [OTU_b], Jaccard [OTU_j], weighted unifrac [OTU_w] and unweighted unifrac [OTU_u]) and Bray-Curtis distances of KO and KEGG pathway (Level3) tables. The R package “corrplot” (ver. 0.84) was taken to visualize these Mantel correlation values with FDR-adjusted *P* < 0.05.**

**Supplementary Figure 8. Spearman correlations between life stage and RBT factors, α diversity indices, and relative abundances of taxonomic (i.e., sex or population-biased taxa) and functional markers (i.e., sex or population-biased KEGG pathways). The R package “corrplot” was taken to visualize these Spearman correlation values with FDR-adjusted *P* < 0.05.**
