# Supplementary figures and images for: Decisive Effects of Life Stage on the Gut Microbiota Discrepancy Between Two Wild Populations of Hibernating Asiatic Toads (Bufo gargarizans)
Source: Front Microbiol. 2021 Aug 3;12:665849. doi: 10.3389/fmicb.2021.665849 (PMC8369469; doi:10.3389/fmicb.2021.665849)

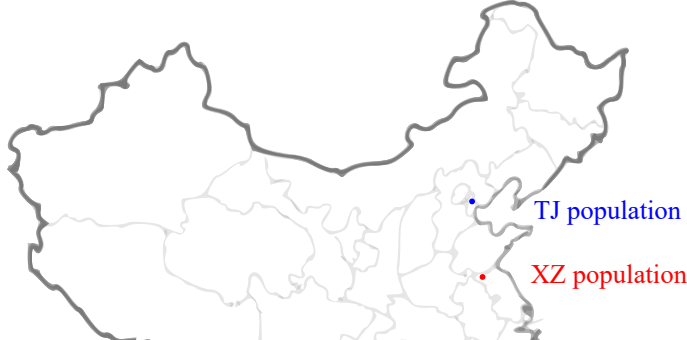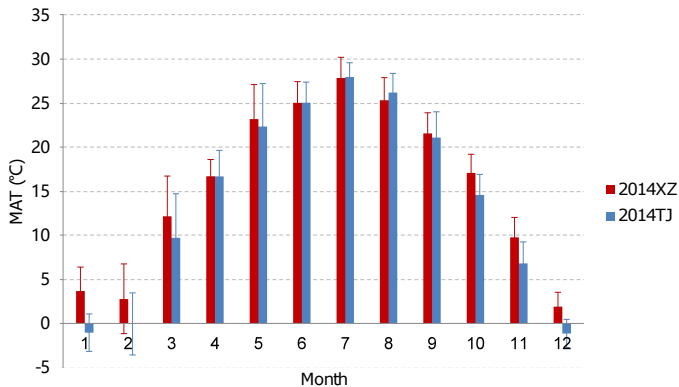

Supplement: Supplementary file 1 [file Data_Sheet_1.zip › Supplementary Material/Supplementary Figure 1.PDF]

Spearman correlations among iterative rarefied OTU tables

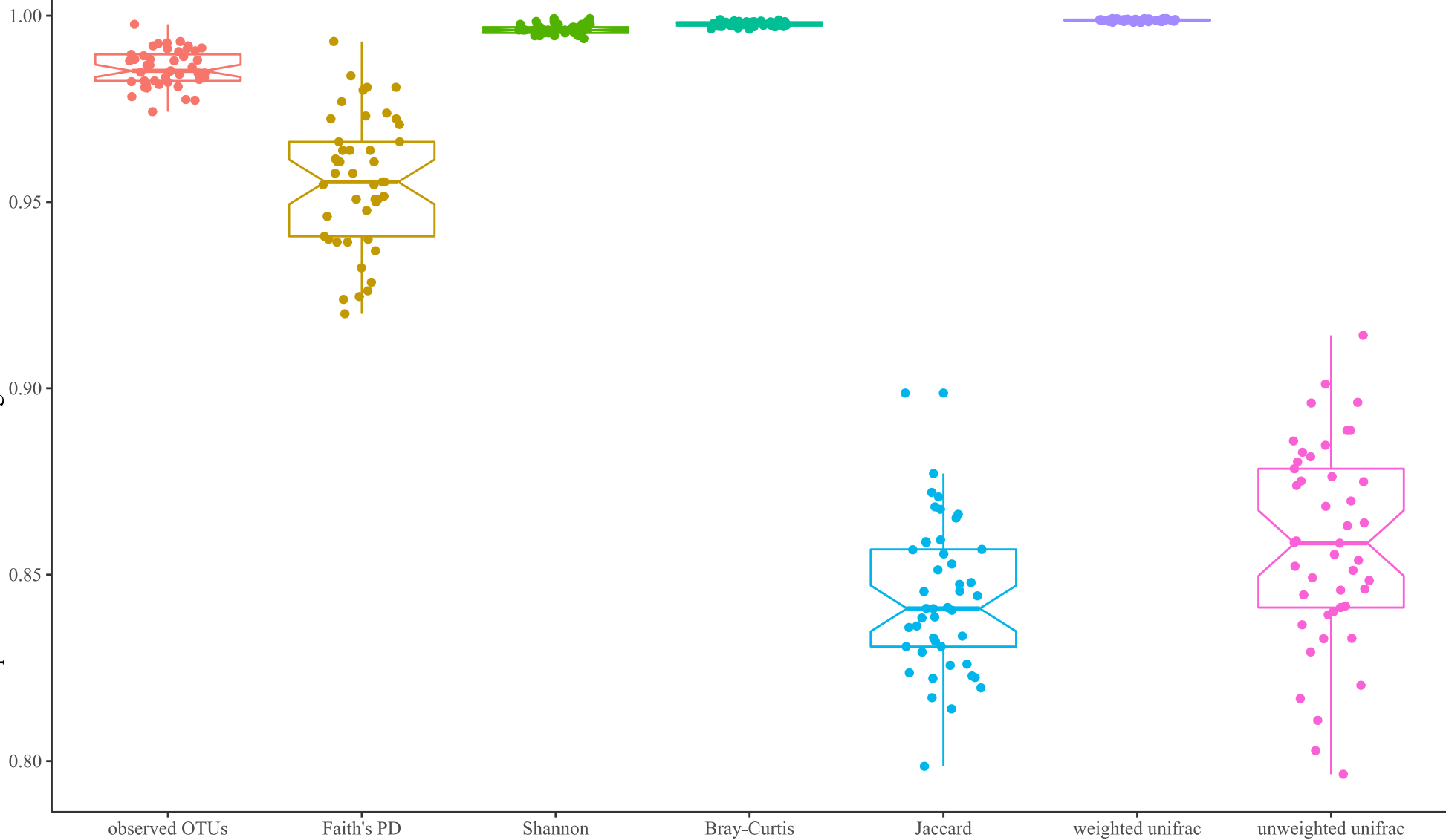

Supplement: Supplementary file 1 [file Data_Sheet_1.zip › Supplementary Material/Supplementary Figure 2.PDF]

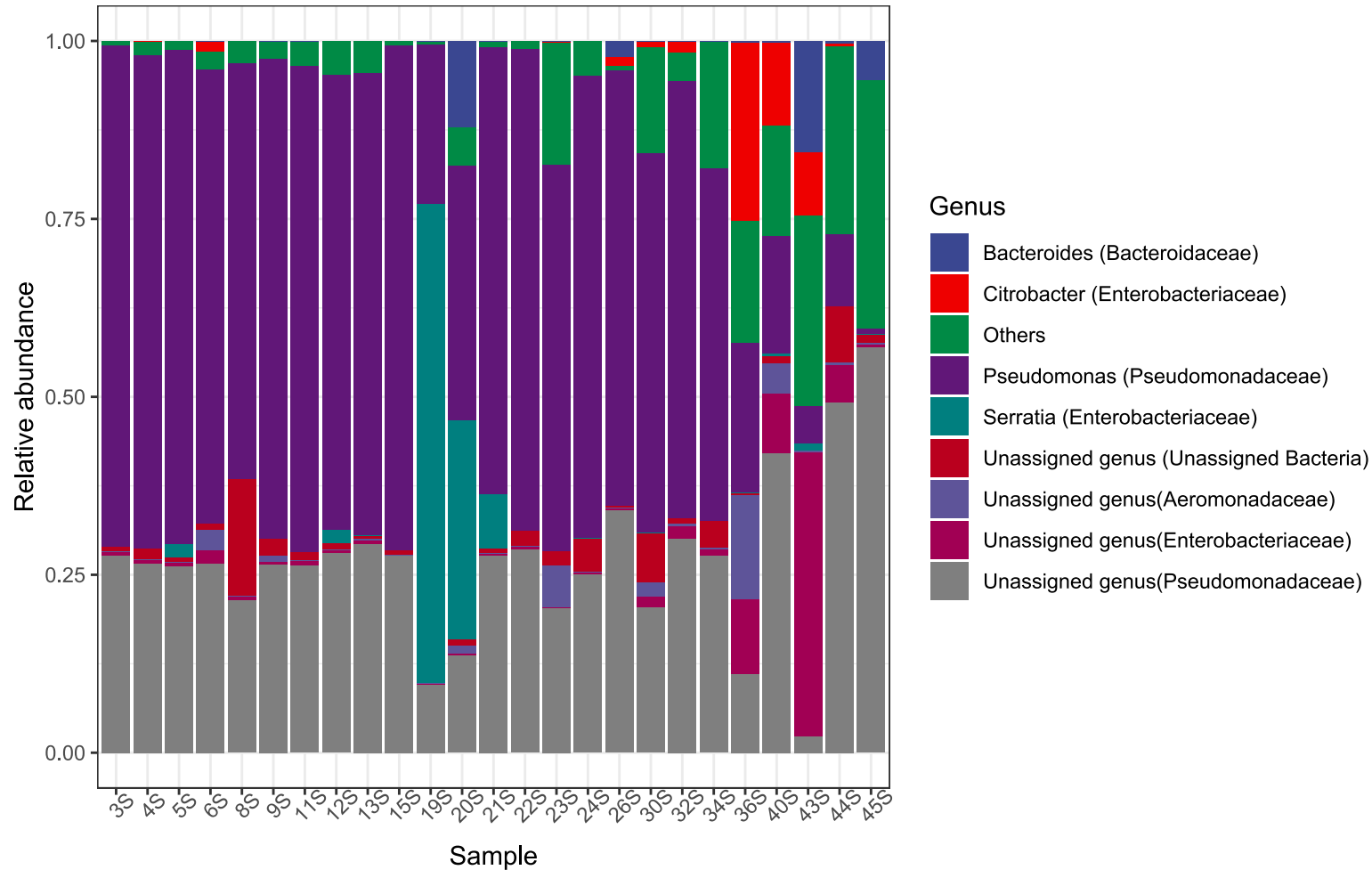

Supplement: Supplementary file 1 [file Data_Sheet_1.zip › Supplementary Material/Supplementary Figure 3.PDF]

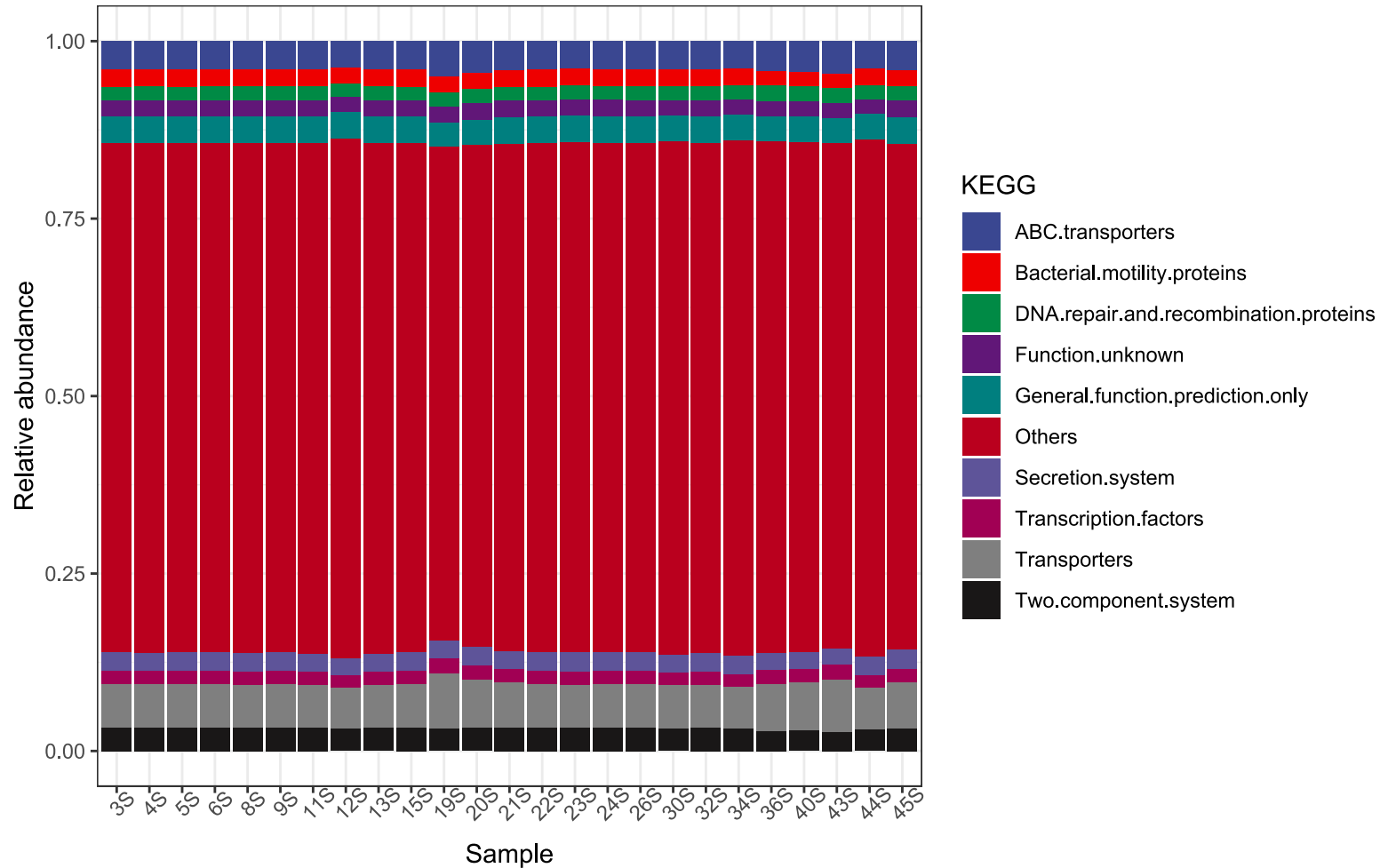

Supplement: Supplementary file 1 [file Data_Sheet_1.zip › Supplementary Material/Supplementary Figure 4.PDF]

Figure S5

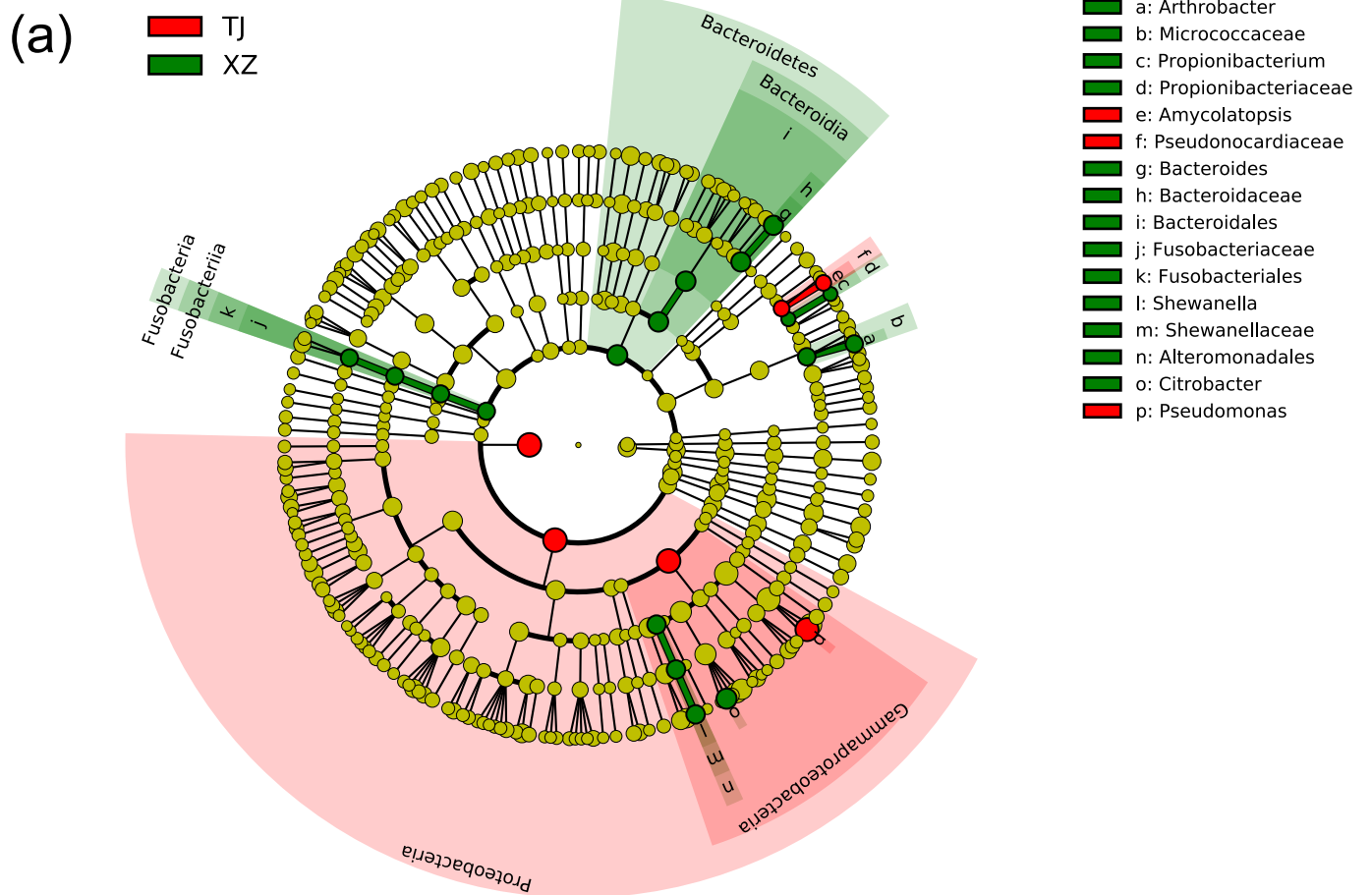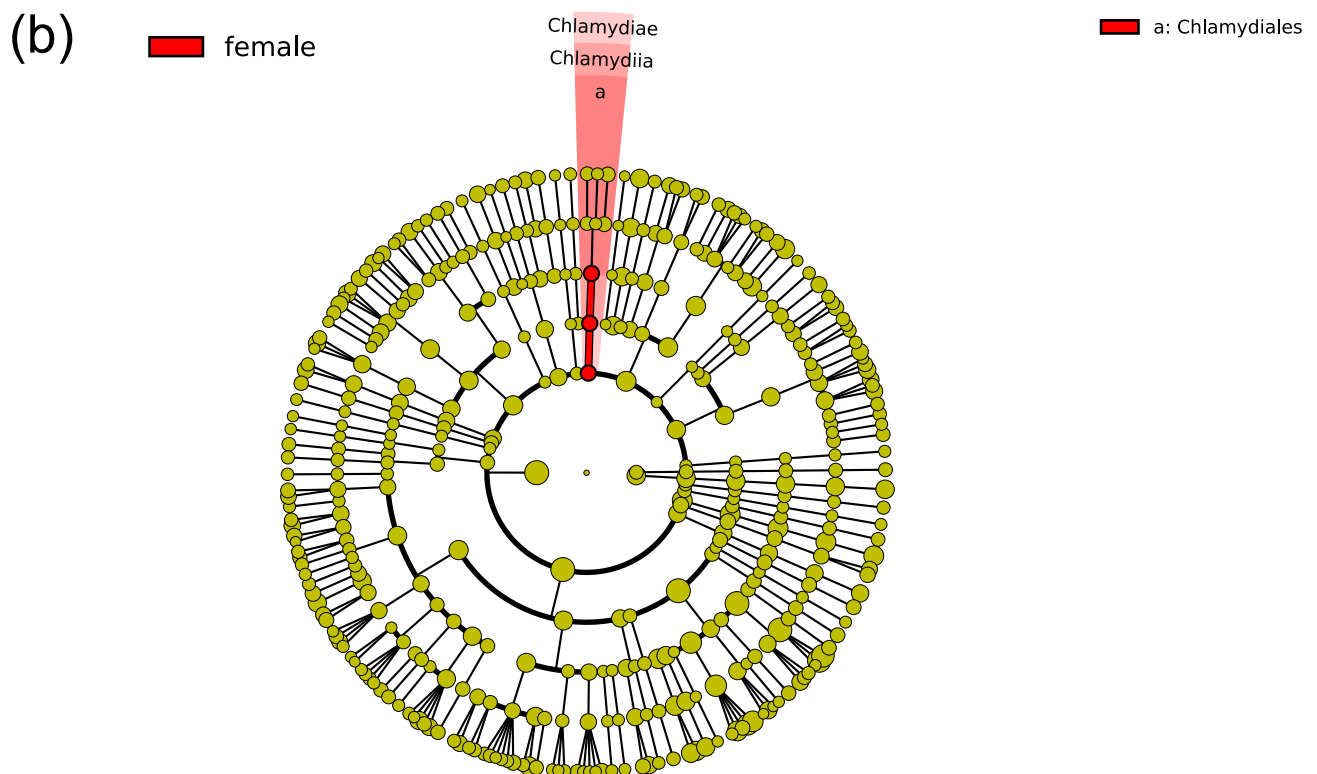

Supplement: Supplementary file 1 [file Data_Sheet_1.zip › Supplementary Material/Supplementary Figure 5.PDF]

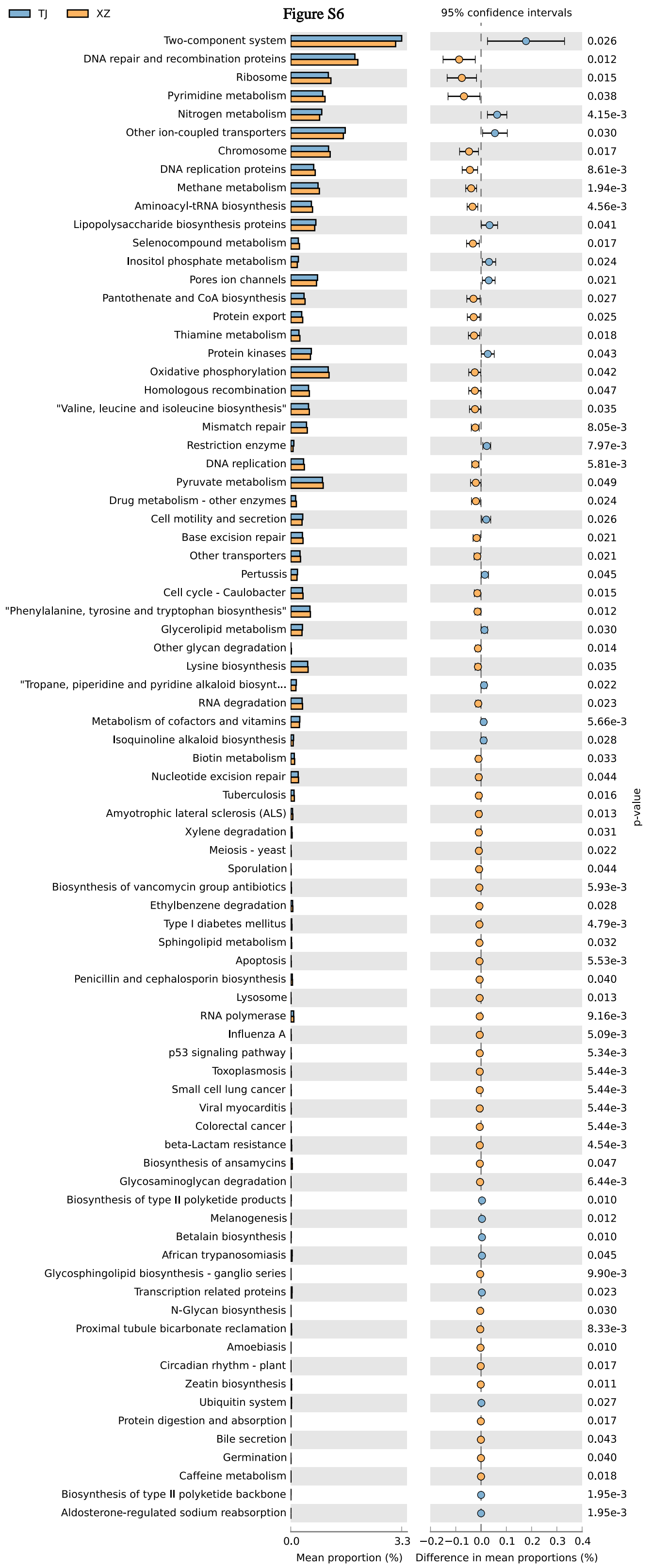

Supplement: Supplementary file 1 [file Data_Sheet_1.zip › Supplementary Material/Supplementary Figure 6.PDF]

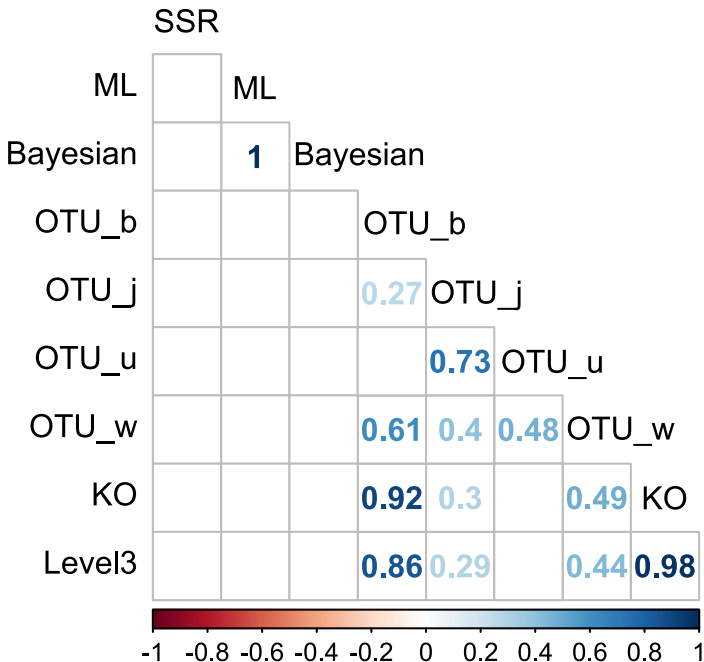

Supplement: Supplementary file 1 [file Data_Sheet_1.zip › Supplementary Material/Supplementary Figure 7.PDF]

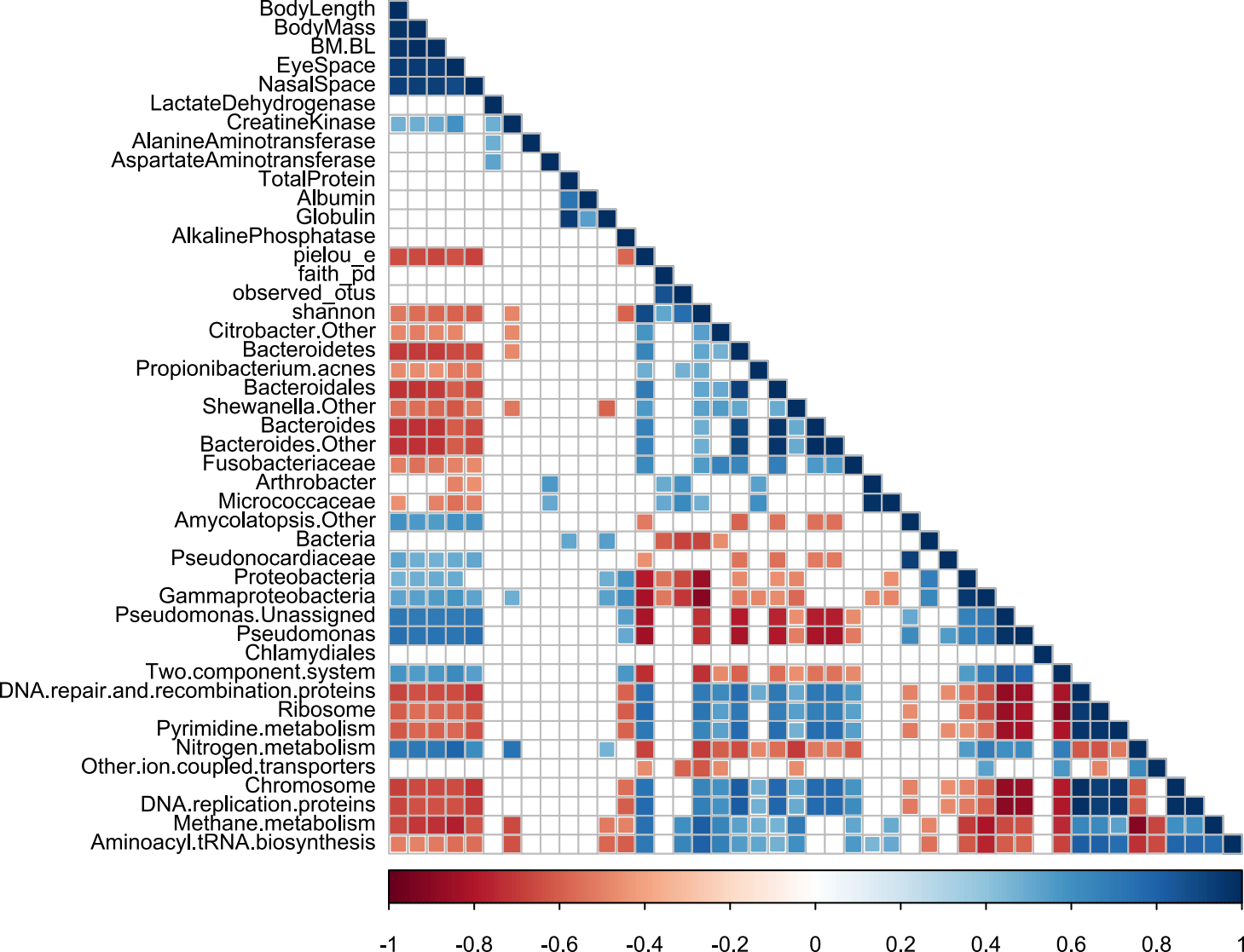

Supplement: Supplementary file 1 [file Data_Sheet_1.zip › Supplementary Material/Supplementary Figure 8.PDF]
